# Mitigated viral myocarditis in A/J mice by the immunoproteasome inhibitor ONX 0914 depends on inhibition of systemic inflammatory responses in CoxsackievirusB3 infection

**DOI:** 10.1007/s00395-021-00848-w

**Published:** 2021-02-01

**Authors:** Carl Christoph Goetzke, Nadine Althof, Hannah Louise Neumaier, Arndt Heuser, Ziya Kaya, Meike Kespohl, Karin Klingel, Antje Beling

**Affiliations:** 1 Charité–Universitätsmedizin Berlin, Corporate Member of Freie Universität Berlin, Humboldt-Universität zu Berlin, and Berlin Institute of Health (BIH), Institute of Biochemistry, Charitéplatz 1, 10117 Berlin, Germany; 2 Deutsches Zentrum für Herz-Kreislauf-Forschung (DZHK), Partner Side Berlin, Berlin, Germany; 3 Department of Pediatrics, Division of Pulmonology, Immunology and Critical Care Medicine, Charité–Universitätsmedizin, Berlin Corporate Member of Freie Universität Berlin, Humboldt-Universität zu Berlin, and Berlin Institute of Health (BIH), Berlin, Germany; 4 Animal Phenotyping Platform, Max-Delbrueck-Center for Molecular Medicine, Berlin, Germany; 5 Cardiopathology, Institute for Pathology and Neuropathology, University Hospital Tuebingen, Tuebingen, Germany; 6 German Rheumatism Research Center (DRFZ), Leibniz Association, Berlin, Germany; 7 German Federal Institute for Risk Assessment, Berlin, Germany; 8 Berlin Institute of Health, Berlin, Germany; 9 Universitätsklinikum Heidelberg, Medizinische Klinik für Innere Medizin III: Kardiologie, Angiologie und Pneumologie, Heidelberg, Germany; 10 Deutsches Zentrum für Herz-Kreislauf-Forschung (DZHK), Partner Side Heidelberg, Heidelberg, Germany

**Keywords:** Infection, Proteasome, Inflammation, Cytokine, Myocarditis

## Abstract

A preclinical model of troponin I-induced myocarditis (AM) revealed a prominent role of the immunoproteasome (ip), the main immune cell-resident proteasome isoform, in heart-directed autoimmunity. Viral infection of the heart is a known trigger of cardiac autoimmunity, with the ip enhancing systemic inflammatory responses after infection with a cardiotropic coxsackievirusB3 (CV). Here, we used ip-deficient A/J-LMP7^−/−^ mice to investigate the role of ip-mediated effects on adaptive immunity in CV-triggered myocarditis and found no alteration of the inflammatory heart tissue damage or cardiac function in comparison to wild-type controls. Aiming to define the impact of the systemic inflammatory storm under the control of ip proteolysis during CV infection, we targeted the ip in A/J mice with the inhibitor ONX 0914 after the first cycle of infection, when systemic inflammation has set in, well before cardiac inflammation. During established acute myocarditis, the ONX 0914 treatment group had the same reduction in cardiac output as the controls, with inflammatory responses in heart tissue being unaffected by the compound. Based on these findings and with regard to the known anti-inflammatory role of ONX 0914 in CV infection, we conclude that the efficacy of ip inhibitors for CV-triggered myocarditis in A/J mice relies on their immunomodulatory effects on the systemic inflammatory reaction.

## Introduction

Enteroviruses (EV), members of the *Picornaviridae* family, comprise a genus of positive-sense single-stranded RNA viruses that are implicated in a broad range of clinical manifestations. For EV-associated health problems such as pancreatic failure, cardiovascular collapse, hepatitis and encephalitis, neither vaccination nor effective antiviral drugs are available [50, 52]. The pressure to develop safe and effective anti-EV drugs is acute, particularly for newborn infants and young children, who, during periods of high EV prevalence, are at risk for life-threatening septic syndromes. CoxsackievirusB3 (CV), belonging to the genus EV, is a clinically relevant and well-studied human pathogen that, in addition to an initial wave of systemic inflammatory responses, can cause a second wave of viral injury targeting the heart and leading to myocarditis with a potentially severe outcome. CV is a pathogen known to cause viral myocarditis in North America and Europe [18]. Infection of laboratory mouse strains with cardiotropic CV reflects the disease in man, including its biphasic course, to a remarkable extent, with evidence for a contribution of specific viral proteins, such as the viral protease 2A, in triggering cytoskeletal protein disruption and shutting down Cap-dependent protein translation in cardiomyocytes [4, 53]. CV exploits the cellular machinery to promote its own replication and thereby disturbs the integrity of infected cells in the heart.

In addition to direct virus-triggered pathology, cardiac dysfunction in viral myocarditis involves inflammatory processes, initially induced by virus replication intermediates activating intracellular pattern recognition receptors (PRR), which help the host prevent uncontrolled virus replication and tissue damage [3, 45, 51]. Concomitant to these innate immune responses, classical pro-inflammatory immune action follows, comprising the infiltration of NK cells and monocytes/macrophages, as well as to a minor extent B and T cells, all assumed to dampen and control virus-triggered pathology [27]. Although there is considerable evidence for a protective contribution of such an immune reaction, there is also consensus that the inflammatory process triggered by virus infection of heart tissue actually drives cardiac damage responses, leading to disarranged cardiac cells, fibrotic tissue repair and ventricular dilation [14]. It was once thought that the presence or the persistence of virus genomes in the human heart determines disease outcome in viral myocarditis, but that idea has since been discarded [19]. In fact, the abundance of infiltrating leukocytes is an independent risk factor for the pathologic progression of acute myocarditis into long-term cardiac dysfunction [30]. The majority of the accumulated leukocytes in inflamed heart tissue of CV-infected mice are CD11b^+^ monocytes and macrophages [35, 40], with infiltration of these immune cells being driven by respective chemotactic cytokines [2]. Others and we demonstrated that particularly those infiltrating myeloid cells contribute to acute and chronic functional impairment in inflammatory heart disease [24, 35]. Another explanation for how virus-induced inflammation results in ongoing inflammatory responses in heart tissue is the fact that acute viral myocarditis can trigger a loss of self-tolerance against cardiac proteins, leading to heart-directed autoimmunity [41]. The underlying immune pathways can be investigated in mouse models of experimental autoimmune myocarditis (AM), where administration of cardiac myosin, troponin I or their pathogenic epitopes, in combination with an adjuvant, induces a cardiac damage response, mimicking central aspects of inflammatory cardiomyopathy in humans [16, 20]. Interestingly, in AM mouse models, infiltrating CD11b^+^ monocytes and macrophages, or the chemotactic molecules produced by these and other immune cells, are also central for disease manifestation [33, 35, 54]. Human monocytes, the main producers of pro-inflammatory cytokines, not only govern inflammation and pro-fibrotic tissue injury in AM, they can also guide the expansion of autoreactive CD4^+^ T cells and their differentiation into Th17 cells, the later promoting cardiac autoimmunity [5, 39].

We have previously demonstrated that the immunoproteasome (ip), the most abundant proteasome isoform in immune cells, regulates the production of pro-inflammatory and chemotactic cytokines by monocytes/macrophages, the activation and differentiation of autoreactive CD4^+^ T cells, as well as cardiac inflammatory responses, both in viral myocarditis and in troponin I-induced AM [2, 12]. The ip is a multi-subunit, barrel-shaped proteolytic complex, where a gated pore within the two outer alpha rings of the complex controls the accessibility of degradation-prone protein substrates to the catalytic center in the interior of the two-fold symmetric chamber. The three β-subunits, β1i (LMP2), β2i (Mecl-1) and β5i (LMP7) [1], in the inner cavity of the complex execute peptide hydrolysis, with each subunit having a cleavage site preference for specific amino acids. Particularly the cleavage-site usage [36] and to a lesser extent the qualitative cleavage site properties [29] demarcate this proteasome isoform from its standard proteasome counterpart, the main isoform found in somatic cells. Both the spatial heterogeneity of proteasome isoforms in distinct cellular compartments and the pro-inflammatory function of the ip in preclinical models of autoimmunity promoted research activities aiming at precisely targeting of the proteasome isoform in immune cells [7, 22, 37, 47, 49]. ONX 0914, a well-studied irreversible epoxyketone inhibitor that blocks virtually all ip subunits to a varying degree [8, 40], and the LMP7-selective dipeptide inhibitor DPLG3 [47] are examples of such accomplishments and both ONX 0914 and DPLG3 can suppress adverse inflammatory tissue damage of the heart [2, 12, 49]. The portfolio of protective features, accomplished by selective inhibition of the ip, encompasses altered T cell activation/differentiation, promoting the survival of fewer Th17 and more regulatory T cells [12, 26, 49], and elevated expression levels of inhibitory immune checkpoint molecules, such as PD-1 [12, 49]. Undisputedly, these aspects are protective in instances like cardiac autoimmunity or allograft rejection after heart transplantation.

However, the contribution of such processes of adaptive immunity to cardiac inflammation, as rapidly evolving as they are after invasion of cardiotropic CV to the heart, is unclear. Our previous study investigated CV myocarditis in A/J mice and we demonstrated that ONX 0914 exerted rapid effects, yielding a profound suppression of the early systemic inflammatory cytokine response that we attributed to impaired signaling processes in myeloid-derived monocytes/macrophages [2]. Whether, however, the improved cardiac output and attenuated myocardial injury that we found in the ONX 0914 group reflects a systemic anti-inflammatory or a more cardio-selective reaction is unclear. Here, we tackled the question of whether the ip can specifically influence viral myocarditis in A/J mice. This would commence in a period after the first wave of viral infection has damaged the pancreas and liver, thereby establishing systemic inflammation and setting up replication of the virus in the heart. We used A/J-LMP7^−/−^ mice to discriminate processes of acute systemic inflammatory responses, such as PRR-triggered pro-inflammatory cytokine production in monocytes/macrophages, known to be unaffected by LMP7 ablation [11, 15, 37], from those of cardiodepressive adverse immune responses present in LMP7^−/−^ mice [7, 9, 12, 37]. In an alternative approach, we selectively inhibited the ip with ONX 0914 during the second wave of viral infection, targeting the heart.

## Materials and methods

### Mice

Original mating pairs of the A/J wild-type mouse strain were purchased from Harlan Winkelmann (A/J). LMP7^−/−^ mice, originally generated by Fehling et al. [17] and provided to our group by the Steinhoff laboratory on a C57BL/6 background, were backcrossed into an A/J background for at least seven generations, using speed congenics. The offspring of heterozygous A/J-LMP7^−/+^ breeding pairs generated LMP7^−/−^ and LMP7^+/+^ wild-type littermate controls (F8). For ONX 0914 treatment in A/J mice, in the majority of experiments, larger cohorts of male A/J mice were purchased from Envigo and mice were allowed to settle for at least 1 week prior to virus inoculation or ONX 0914 treatment. Male animals (− 8 weeks) were injected intraperitoneally (i.p.) with 10^4^ PFU of a cardiotropic variant of CVB3 Nancy strain [31]. All mice were kept at the animal facilities of the Charité University Medical Center. For the therapeutic ONX 0914 treatment, mice were injected subcutaneously with 10 mg/kg body weight (BW) ONX 0914 or the vehicle Captisol daily from days 3 to 7. On the final day of each infection study, after echocardiography, organs were collected for further analysis and immediately frozen in liquid nitrogen. Organs were stored at − 80 °C. Heart tissue was fixed in HistoFix (1xPBS, 4% ROTI^®^Histofix) overnight and embedded in paraffin. To visualize cardiac infiltration, cross sections were stained with hematoxylin and eosin and judged by a pathologist as described elsewhere [45]. Whole blood was centrifuged at 4 °C and 10,000 rcf for 15 min to separate serum, which was then collected and stored at − 80 °C.

This study was carried out in accordance with the recommendations in the Guide for the Care and Use of Laboratory Animals of the German Animal Welfare Act, which is based on the directive of the European Parliament and the Council of Europe Convention for the Protection of Vertebrate Animals used for Experimental and other Scientific Purposes (ETS123). This study was approved by the local authorities for animal welfare in Berlin (permit numbers: G0054/18, G 0274/13, G103/18). All efforts were made to minimize suffering.

### Echocardiography

For echocardiography, mice were anesthetized with 1.5–2% isoflurane and kept warm on a heated platform. Temperature and ECG were monitored continuously. Cardiac function and morphology were assessed with a VisualSonics Vevo 3100 High-Frequency Imaging System using a high-resolution (44 MHz) transducer. Standard imaging planes, M-mode, and functional calculations were obtained. For the parasternal long axis LV trace, the average systolic or diastolic volume in B-Mode is based on the rotational volume of the LV trace at systole or diastole around the long axis of the spine. The parasternal long-axis view of the left ventricle (LV) was used to guide calculations of ventricular dimensions (M-mode), volumes (B mode; LV vol; *d* = LV trace end-diastolic; LV vol; *s* = LV trace end-systolic; stroke volume using the formula  =  v; − v;) and left ventricular ejection fraction (B mode using the formula  = 100 × ( v; − v; v; )). M-mode echocardiographic images were recorded at the level of the papillary muscles from the parasternal short-axis view. All measurements were performed by the Animal Phenotyping Platform of the Max-Delbrück-Center for Molecular Medicine in Berlin.

### Cell culture, isolation of immune cells from mouse tissue

For the analysis of bone marrow cells, flushed bone marrow was passed through 40 μm cell strainers, centrifuged, and resuspended with RPMI. Splenocytes were prepared by passing spleen tissue trough a 70 μm cell strainer (BD Biosciences). After a wash step with 1 × PBS, RBC lysis was performed 2–3 times by incubating in 0.83% ammonium chloride (NH_4_Cl) for 3–5 min at room temperature. Cells were recovered by centrifugation (10 min, 310*g*), re-suspended in FACS buffer, and chilled on ice until flow cytometry was carried out.

For heart tissue, after extraction of whole blood the heart was flushed with 15 mL PBS, removed and washed again in PBS. An amount of heart tissue defined by weight was minced in RPMI 1640 medium (Biochrom) containing 10% (v/v) fetal calf serum (FCS) (Biochrom), 1% (v/v) penicillin/streptomycin (Pan Biotech), 30 mM HEPES, 0.1% (w/v) collagenase type 2 (Worthington) and 0.015% (w/v) DNase I (Sigma-Aldrich). Tissue digestion was carried out by incubating at 37 °C for 30 min while shaking at 800 rpm. To ensure the cells did not clump together, 10 mM EDTA was added, the cells were washed with PBS, and were passed through a 70 μm cell strainer (BD Biosciences). Cells were recovered by centrifugation (10 min, 310*g*), re-suspended in FACS buffer, and chilled on ice until flow cytometry was performed.

### Proteasome inhibitor ONX 0914

For subcutaneous administration in mice, ONX 0914 (Cayman Chemicals) was dissolved at a concentration of 1–2 mg/mL in an aqueous solution of 1 mg/mL Captisol (Ligand Pharmaceuticals) and sodium citrate at a pH of 3.5. Aliquots of both ONX 0914 and Captisol were stored at − 20 °C.

### Flow cytometry

Equal numbers of splenocytes or cells purified from 15 mg heart tissue were incubated (20 min at 4 °C) in FACS buffer (1xPBS, 2% FCS, 2 mM EDTA) containing an anti-mouse Fc receptor blocking reagent (1:50; Miltenyi Biotec). Afterwards, fluorochrome-conjugated antibodies against various surface markers were added and incubated for at least 20 min at 4 °C while protected from light. The following antibodies were purchased from BD Bioscience: CD8α (FITC; clone 53-6.7), B220 (PE; clone RA3-6B2), CD90.2/Thy-1.2 (PE; clone 53-2.1), TER-119 (PE; clone TER-119), CD11b (PE-CF594; clone M1/70), CD4 (V500; clone RM4-5), CD8α (Pacific Blue™; clone 53.6.7). CD49b (PE; clone DX5), CD44 (PE; clone IM7) and CD3 (APC; clone 2-C11) were purchased from eBioscience. CD45.2 (Brilliant Violet 711™; clone 104), Ly6G (PerCP/Cy5.5; clone 1A8), Ly6C (Pacific Blue™; clone HK1.4), CD11c (Brilliant Violet 510™; clone N418), F4/80 (APC; clone BM8), CD3 (PerCP/Cy5.5; clone 145-2C11), B220 (FITC; clone RA3-6B2), CD19 (APC; clone 6D5) were purchased from BioLegend. After several wash steps with FACS buffer (centrifugation: 3 min at 300*g*), cells were re-suspended in 150 μL of the fixable viability dye eFluor 780 (eBioscience), diluted 1:1.000 in PBS and incubated for 30 min on ice protected from light. After serial wash steps with PBS followed by fixation in FACSFix (1x PBS, 2% RothTMHistofix), cells were acquired on either a FACS Symphony flow cytometer (BD Biosciences). Data were analyzed using FlowJo v10.0 software (Tree Star). To quantify total cell numbers in heart tissue, 123 count eBeads (eBioscience) were used according to the manufacturer’s protocol. Reported numbers were normalized for the weight of total hearts yielding the number of cells per mg tissue. Monocytes were identified by positive gating for CD45, CD11b and exclusion of Ly6G, CD11c and F4/80-positive cells in addition to lineage negative staining (B220, CD90.2, CD49b and Ter-119) as recently described [23]. Expression of Ly6C was used to discriminate between patrolling and inflammatory monocytes.

### RNA isolation and quantitative real-time PCR (qPCR)

RNA was isolated using TRIzol^®^ (Ambion) method according to the manufacturer’s instructions. Remaining DNA was removed by digestion with DNAse I (Invitrogen) at 37 °C for 15 min followed by enzyme deactivation at 65 °C for 10 min. 250–1000 ng RNA were reverse transcribed with MLV Reverse Transcriptase (Promega) in combination with random hexamer primers (Roche). TaqMan^®^ PCR was performed using primers and probes of TaqMan^®^ gene expression assays (Life Technologies) as well as the following combinations of primers and probes: murine HPRT fw: 5′-ATC ATT ATG CCG AGG ATT TGG AA-3´, rev: 5′-TTG AGC ACA CAG AGG GCC A-3′, probe: 5′FAM- TGG ACA GGA CTG AAA GAC TTG CTC GAG ATG -3′TAMRA; CV fw: 5′-CCC TGA ATG CGG CTA ATC C-3′, rev: 5′-ATT GTC ACC ATA AGC AGC CA-3′ probe: 5′-FAM-TGC AGC GGA ACC G -MGB3′. qPCR was conducted on a StepOnePlus™ real-time PCR system. TaqMan^®^ assays for *HPRT* served as endogenous controls and were used to calculate relative expression using the ΔC(t) or ΔΔC(t) method.

### Quantification of infectious viral particles

Plaque assays were performed on sub-confluent monolayers of green monkey kidney cells incubated with serial tenfold dilutions of cell culture supernatant or supernatant from homogenized mouse tissue. After incubation at 37 °C for 1 h, supernatants were removed and monolayers were overlaid with DMEM containing 1 mM pyruvate, 2.5% FCS, 3.75% NaHCO_3_ and 0.6% agar. 2 days later cells were fixed with 75% methanol/25% acetic acid and stained with 0.25% crystal violet solution to count virus plaques.

### Western blot analysis

For SDS-PAGE, cells or tissue were lysed using 8 M urea buffer containing 1% Triton, 0.1% SDS, 20 mM HEPES, 8 mM EDTA, 2 mM EGTA, 50 mM sodium fluoride, 5 mM sodium pyrophosphate, 2 mM sodium orthovanadate, 1 mM TCEP and complete protease inhibitor cocktail (Roche). Western blot analysis was performed according to standard protocols. The following primary antibodies were used: β-actin (C4, Merck), α4 (K378/1, lab stock), α6 (K379, lab stock), β1(K43, lab stock), β5 (Abcam 3330), LMP2 (Abcam 3328) and LMP7 (K63, lab stock). Secondary IRD680CW- or IRDye800CW-labeled antibodies were detected by the Odyssey CLx infrared imaging system (Li‐Cor Biosciences). Densitometric analysis was performed using Image Studio™ (Li‐Cor Biosciences) and values were normalized to internal loading controls.

### Statistics

Statistical analysis of the data was performed in GraphPad Prism v7.00 and v8.00 for Windows (GraphPad Software). All data is plotted as individual points. If not indicated otherwise, data summary are given as mean ± standard error of the mean (SEM). Data was first tested for normal distribution using the D’Agostino-Pearson test. For non-normal distributed data a Mann–Whitney-test was used. Unpaired *t*-tests were used for two group comparisons of normal distributed data. If samples had unequal variances (determined by an *F* test), an unpaired *t* test with the Welch correction was used. For multiple group comparison, unequal variance versions of ANOVA (two-way ANOVA) were used. Survival was test for using a Gehan–reslow–Wilcoxon test. The significance threshold for all tests was set at the 0.05 level.

## Results

### Altered proteasome isoform composition in ip-deficient LMP7^−/−^ mice mediates inflammatory perturbation of cardiac function in CV infection

A comparison of ip-deficient A/J-LMP7^−/−^ mice with their wild-type controls revealed the pro-inflammatory role of the ip for the onset and manifestation of cardiac autoimmunity, with both processes being reversible in wild-type mice by treatment with ONX 0914 [12]. LMP7^−/−^ mice resemble WT mice after ONX 0914 treatment with regard to the relative expression levels of the different ip subunits. They not only show a complete lack of LMP7, but also a profound reduction of Mecl-1 and lower LMP2 expression levels [40, 42]. This defect of intact ip formation in LMP7^−/−^ mice can be attributed to the cooperative assembly of ip complexes [21]. Other than shown for PRR-activated immune cells after ip inhibition by ONX 0914, PRR-triggered cytokine responses, however, are not affected in LMP7^−/−^ immune cells [12, 43]. Based on the coherent phenotype found in both LMP7^−/−^ mice and in wild-type mice after ip inhibition in troponin I AM, we first asked whether the immunosuppressive effects seen in ONX 0914-treated A/J mice during CV infection can be phenocopied in A/J-LMP7^−/−^ mice. If so, it would indicate that ip-dependent control of CV infection might be attributed to an altered adaptive immune response.

**Fig. 1:**
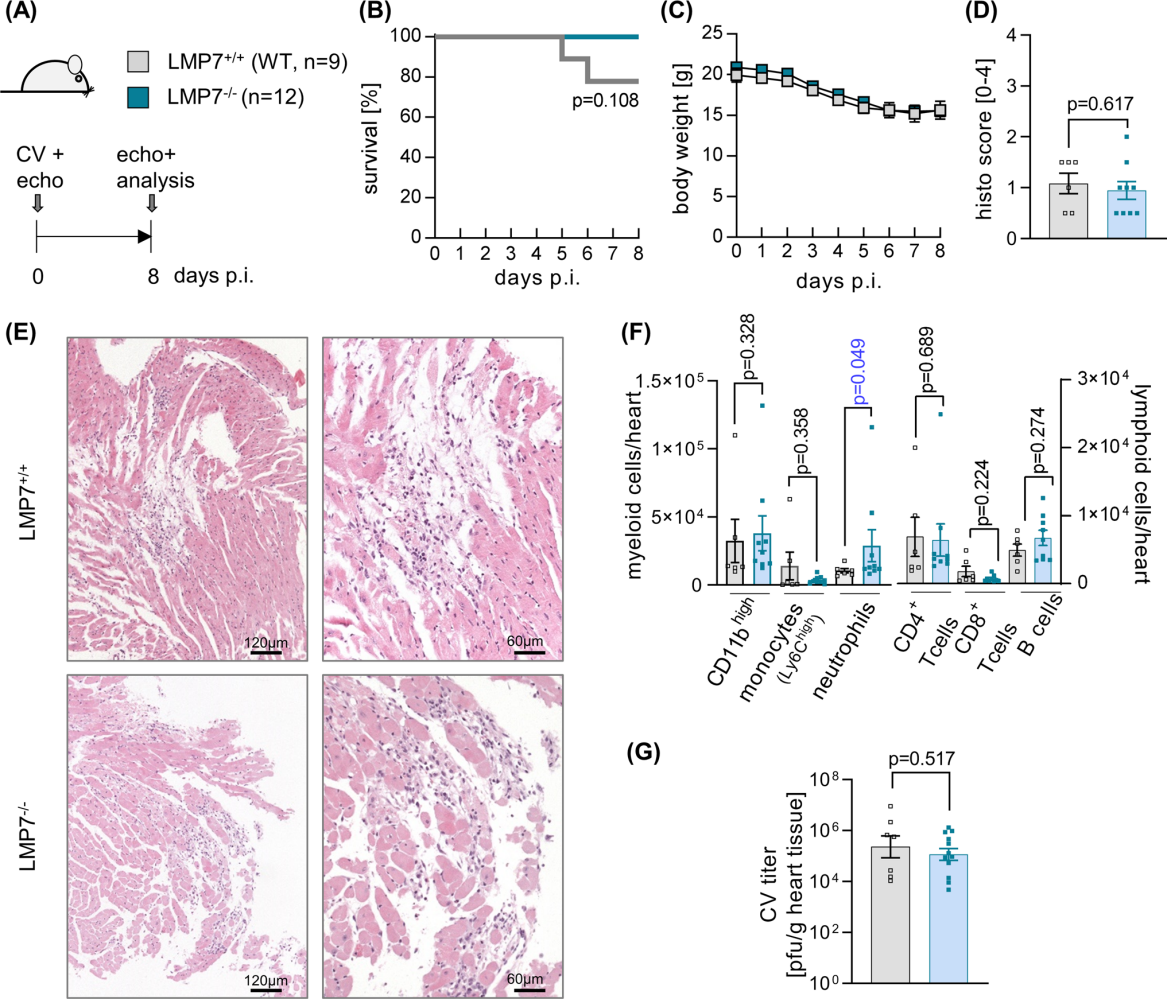
Phenotype of CV infection in ip-deficient LMP7^−/−^ A/J mice. **a** LMP7^+/+^ (wild-type littermate controls, *n* = 9) and age and gender matched LMP7^−/−^ mice (*n* = 12) were infected with 10^4^ pfu of CV Nancy and were analysed on day 8 postinfection (p.i.). **b** Survival and **c** body weight was monitored daily. **d** Formalin fixated and paraffin embedded heart tissue sections were stained using hematoxylin and eosin and myocarditis severity was assessed. **e** Representative micrographs of haematoxylin and eosin stains are depicted for each group. **f** For further quantification of immune cell infiltration into the heart tissue, infiltrating immune cells per total heart were quantified by flow cytometry. **g** Virus concentration in heart tissue was assessed by plaque assay

LMP7^−/−^ mice and their age- and gender-matched LMP7^+/+^ littermate controls were followed after infection with cardiotropic CV Nancy for 8 days, revealing no relevant effect of LMP7 ablation on global health parameters, such as survival or body weight (Fig. 1b, c). Histological scoring of HE-stained heart tissue sections to evaluate myocardial necrosis and inflammation revealed profound myocarditis with similar scores in LMP7^−/−^ mice and their littermate wild-type controls (Fig. 1d, e). Correspondingly, a quantification of infiltrated immune cells in heart tissue by flow cytometry demonstrated an equivalent distribution of CD11b^high^ myeloid cells, with a slightly higher number of neutrophils in LMP7^−/−^ mice. The overall abundance of myeloid and lymphoid immune cells, however, was similar in both groups (Fig. 1f), confirming that the inflammatory heart tissue injury is not influenced by the impaired ip expression in LMP7^−/−^ mice. In addition to immune response-related effects, myocardial tissue injury observed 8 days after CV infection reflects the extent of viral cytotoxicity. To address the question of whether ablation of LMP7 in A/J mice affects the virus concentration during acute myocarditis, we quantified the virus titer in heart tissue by plaque assay and found an equivalent virus load of 5.4 × 10^5^ ± 0.4 × 10^5^ pfu/g in LMP7^+/+^ mice and 5.1 × 10^5^ ± 0.2 × 10^5^ pfu/g in LMP7^−/−^ mice (Fig. 1g).

To investigate whether ablation of LMP7 influences inflammation-triggered perturbation of cardiac function, as shown previously for ONX 0914-treated mice in viral myocarditis [2], we performed echocardiography prior to infection and during the acute phase of myocarditis. In line with our previous data [2, 40], the ejection fraction of the left ventricle was within the range of the values measured in naive mice prior to infection, regardless of LMP7 expression (Table 1). We found a reduced cardiac output, attributed to lower heart rate and an impaired stroke volume due to reduced left ventricular filling, both in LMP7^−/−^ and control mice infected with CV (Table 1). Together, these data reflect that the inflammation-induced perturbation of the cardiac output, known to be mediated by the catalytic activity of the ip [2], can be similarly affected by the compensatory proteasome activity present in ip-incompetent LMP7^−/−^ mice.

**Table 1:** Analysis of cardiac function in LMP7^−/−^ mice during acute CV myocarditis

	LMP7^+/+^ (WT)		LMP7^−/−^	
	Baseline	Day 8	Baseline	Day 8
Heart rate (bpm)	402 ± 23	363 ± 33	429 ± 17	368 ± 20
Trace EF (%)	57.8 ± 2.9	60.6 ± 1.1	57.0 ± 2.3	62.9 ± 2.4
Cardiac output (mL/min)	8.4 ± 0.6	6.3 ± 1.2	9.8 ± 0.6	6.0 ± 0.7*
Stroke volume (µL)	20.9 ± 1.0	16.5 ± 2.0	22.9 ± 0.8	16.1 ± 1.3*
Vol d (µL)	36.2 ± 1.0	27.2 ± 3.2*	40.3 ± 1.0	25.8 ± 2.2*
Vol s (µL)	15.3 ± 1.2	10.7 ± 1.2*	17.4 ± 1.2	9.7 ± 1.2*^,§^
LVID-d (mm)	3.6 ± 0.1	3.0 ± 0.2*	3.7 ± 0.1	3.0 ± 0.1*
LVID-s (mm)	2.5 ± 0.1	2.1 ± 0.1*	2.5 ± 0.1	2.0 ± 0.1*

Echocardiography was performed in LMP7^−/−^ mice and age- and gender-matched littermate controls prior to infection (baseline, day 0) and on day 8 post-infection (*n* = 9 per group). Data shown are mean values ± SEM and were analyzed using repeated measurements two-way ANOVA, followed by Sidak’s multiple comparison test

EF: ejection fraction; bpm: beats per minute; Vol d/s: end-diastolic/-systolic left ventricular volume; LVID-d/s: left ventricular inner dimension at diastole/systole

*Indicates significant differences in the respective treatment group at day 8 compared to this groups’ baseline measurement. There were no differences regarding to gene deficiency for LMP7

**Fig. 2:**
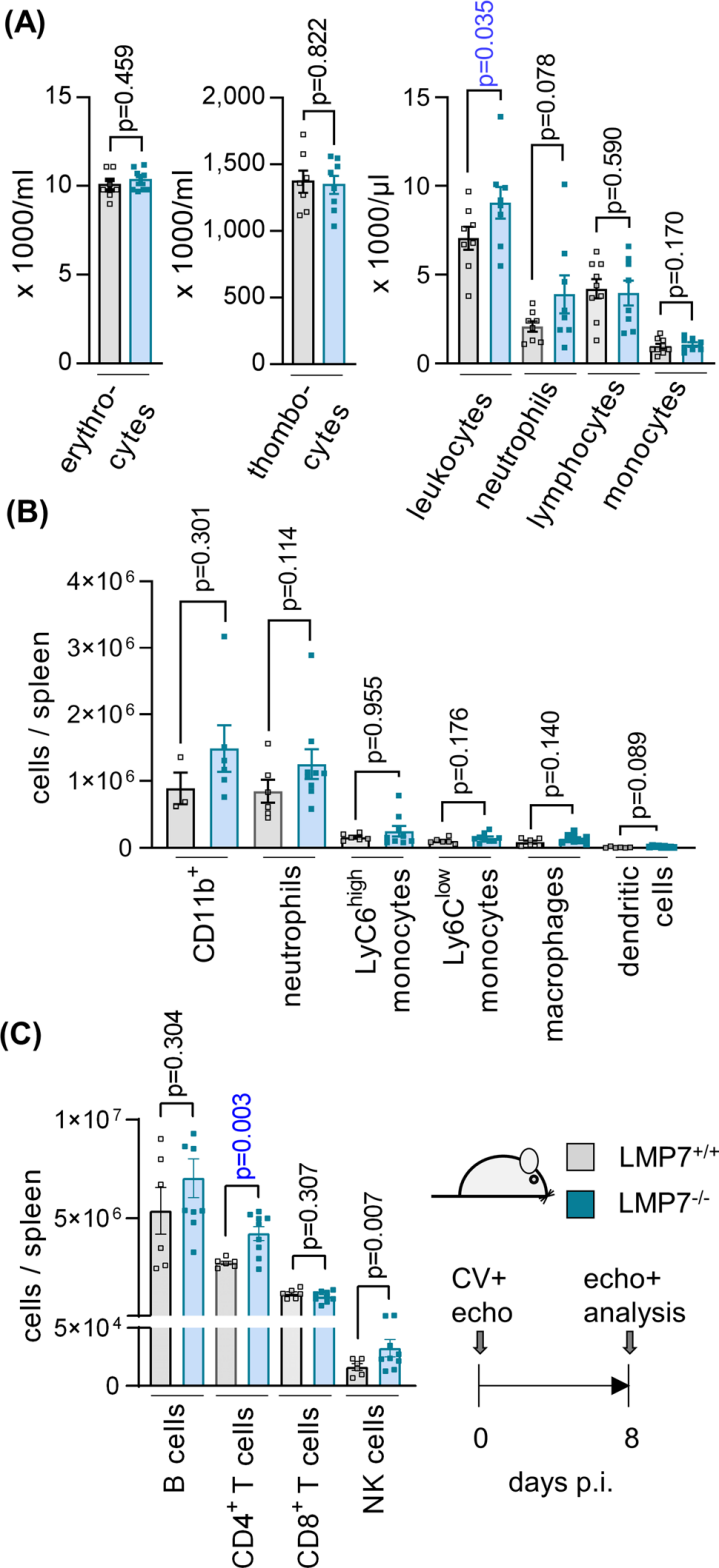
Peripheral immune cell composition in LMP7^−/−^ A/J mice during CV infection. **a** At day 8 p.i., blood was drawn for a peripheral blood count. **b**, **c** At the same time point, spleen tissue was homogenized and single cell suspensions were subjected to immune cell quantification by flow cytometry (*n* = 6 LMP7^+/+^ and *n* = 9 LMP7^−/−^ mice)

Since the modified proteasome activity, inherent in LMP7^−/−^ mice with ip dysfunction [10, 12], apparently neutralizes the immunomodulatory potential of the ip that can be targeted in wild-type mice by inhibitors of this proteasome isoform, we questioned how other immune-related aspects with a specific functional requirement for intact ip proteolysis are influenced in LMP7^−/−^ mice during CV infection. The peripheral blood count in CV infected mice showed a significantly elevated level of leukocytes, and may be showing a trend towards higher neutrophil counts in LMP7^−/−^ mice (Fig. 2a), similar to findings in ONX 0914-treated A/J mice [2]. Spleen tissue, known to feature elevated levels of myeloid cells during CV infection, had a similar distribution of CD11b^high^ immune cells (Fig. 2b). Of note, the abundance of inflammatory monocytes, which can be captured in spleen tissue of CV-infected mice by ip inhibition [2], was similar in both LMP7^−/−^ mice and their littermate controls (Fig. 2b), corresponding to the equal CD11b^high^ cell count in the heart during myocarditis in these groups (Fig. 1f). A quantification of the lymphoid cells in spleen tissue showed a higher CD4^+^ T cell count and elevated numbers for NK cells in infected LMP7^−/−^ mice, with no effect on B cells or CD8^+^ T cells (Fig. 2c). Together, aside from neutrophils, the overall distribution of the immune cells and specifically that of CD11b^+^ myeloid cells in infected mice differs between ip-deficient LMP7^−/−^ mice (Fig. 2) and WT mice with inhibited ip activity [2].

### Altered proteasome isoform composition in ip-deficient LMP7^−/−^ mice allows ip-mediated effects on myeloid immune cells

**Fig. 3:**
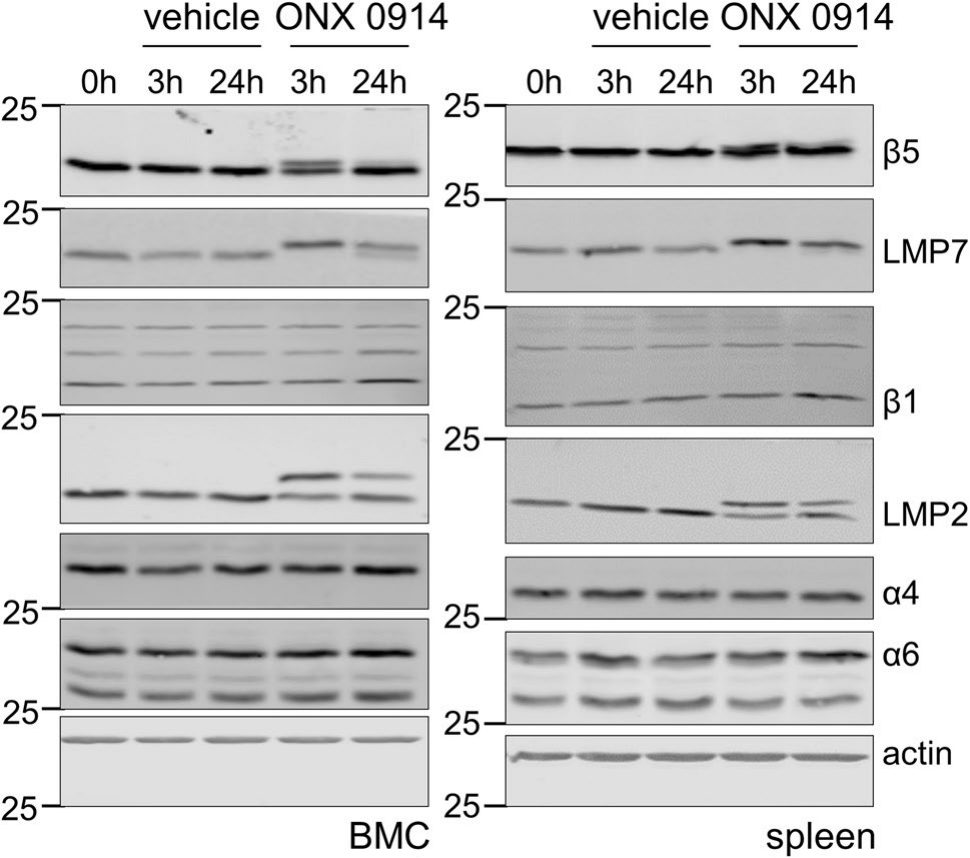
Proteasome inhibition and effects on the expression levels of proteasome subunits by short time ONX 0914 treatment. A/J mice were treated with the vehicle Captisol^®^ or 10 mg/kg BW of the ip inhibitor ONX 0914. After 3 and 24 h, bone marrow cells (BMC) and spleen tissue were removed and protein lysates were subjected to Western blot analysis (*n* = 1 per time point and treatment). Proteasome composition in each sample was analysed using primary antibodies against the α4 and α6 subunits expressed in all proteasome isoforms and the proteolytically active immunoproteasome subunits LMP7 and LMP2 and their respective standard proteasome counterparts β5 and β1. The additional upwards shifted bands indicate a binding and inactivation by ONX 0914 of the respective proteasomal subunits. Actin was used to indicate protein loading

To differentiate the effects of ip-deficiency in LMP7^−/−^ mice from those of ONX 0914-treated WT mice in terms of the cellular composition of the main immune tissue compartments, we compared CD11b^high^ leucocytes in bone marrow, spleen tissue and blood from naive LMP7^−/−^ mice with those of ONX 0914-treated mice. First, we asked how short term treatment with ONX 0914 affects the proteasome composition in immune cell compartments. Figure 3 shows the Western blot-based analysis of proteasome subunits in bone marrow cells (BMC) and spleen, exemplarily performed for the LMP7/β5i, LMP2/β1i, β1, β5, α4 and α6 subunit of ONX 0914-treated A/J WT mice 3 and 24 h after subcutaneous injection of the compound and in comparison to vehicle (Captisol) treatment. A complete upward shift of the LMP7/β5i subunit, reflecting the irreversibly bound ONX 0914 in complex with this subunit, demonstrates that the accessibility of substrates to the active site of LMP7/β5i is blocked. LMP2/β1i is partially affected by ONX 0914 and after 3 h β5 is slightly inhibited, but recovers full activity by 24 h. Importantly, other than what has been reported for LMP7^−/−^ mice [40, 43], using this set-up, ONX 0914 had no effect on the overall proteasome levels or on catalytic subunits of the standard proteasome, as indicated by similar expression levels for α4 and α6, as well as β1 and β5, respectively.

**Fig. 4:**
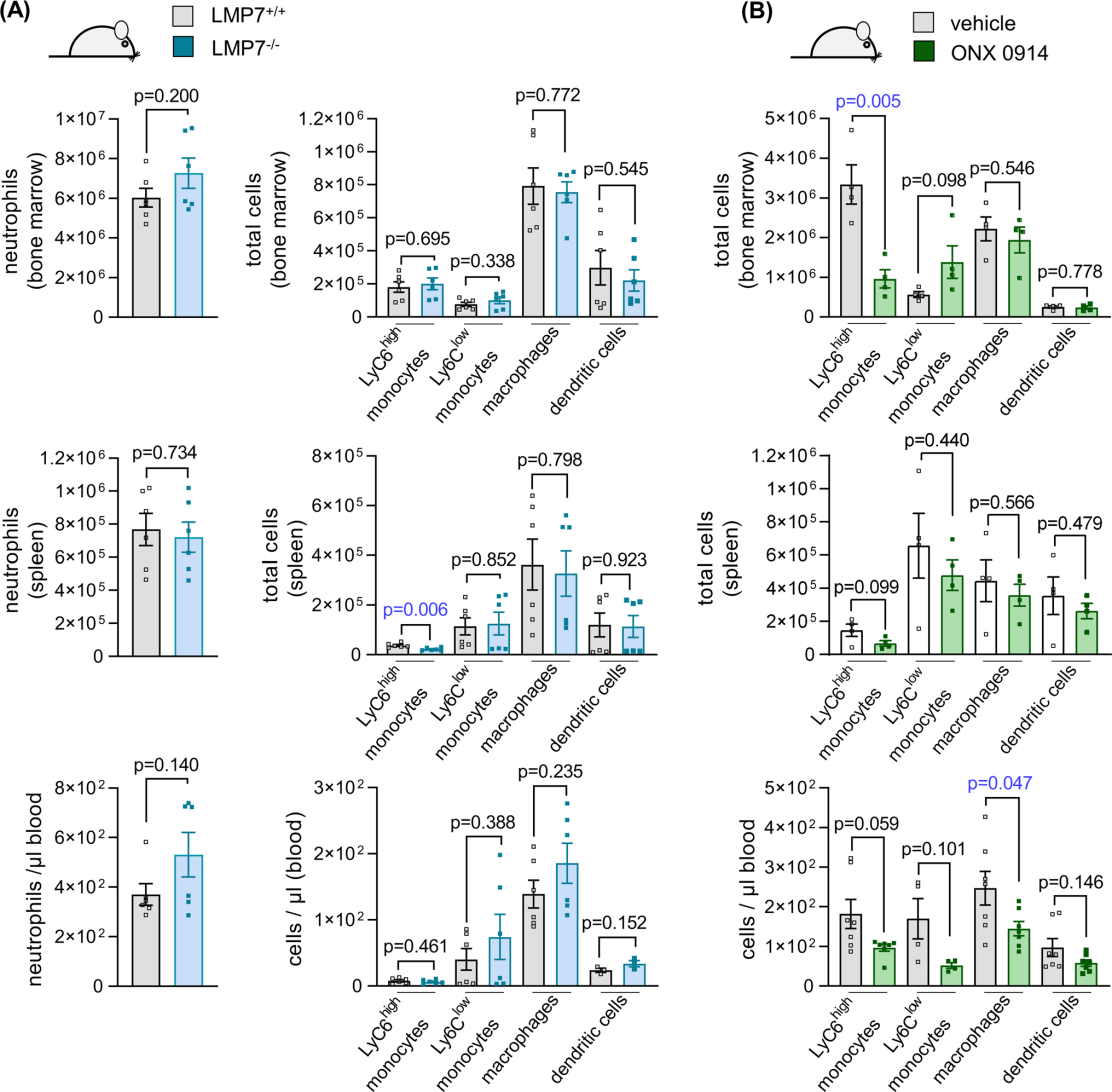
Immune cell phenotyping in naive ip-deficient LMP7^−/−^ A/J mice and ONX 0914 treated A/J mice. **a** Immune cell composition of LMP7^+/+^ and age and gender matched LMP7^−/−^ littermates (*n* = 6 per group) was analysed in the bone marrow, spleen and peripheral blood by flow cytometry. Bone marrow was taken from 2 femurs and 2 tibias of each mouse and total cells were quantified. **b** Similarly, in A/J mice treated with vehicle (*n* = 4) or 10 mg/kg BW ONX 0914 (*n* = 4), immune cell compartments were harvested 18 h later. Immune cells were quantified by flow cytometry in the bone marrow of 2 femurs and 2 tibias, spleen and peripheral blood. The variability between the absolute immune cell abundancies, e.g., for Ly6C^high^ monocytes in bone marrow from WT mice in **a** and sham-treated WT mice in **b**, may be attributed due to heterogenous experimental conditions and slightly diverse counting approaches of the two experimenters for **a** and **b**. A direct comparison between the absolute cell counts in (**a**) and (**b**) is not valid

We then analyzed CD11b^high^ immune cells from age- and gender matched naive LMP7^−/−^ and LMP7^+/+^ A/J mice, as well as ONX 0914 and Captisol-treated A/J WT mice, by flow cytometry. The results depicted in Fig. 4a show that the myeloid immune cell composition is unaffected in naive LMP7^−/−^ mice, with equivalent counts of monocytes, macrophages and dendritic cells in bone marrow, spleen and blood when compared to their WT controls. In contrast, inhibition of the ip by ONX 0914 in naive A/J WT mice reduced the abundance of inflammatory monocytes, showing the most prominent reduction in bone marrow (Fig. 4b). From these experiments, we conclude that the pathophysiological effects controlled by inflammatory monocytes, which are crucial in CV myocarditis [35], might differ a great deal in ip-deficient LMP7^−/−^ and WT mice in which ip proteolysis has been inhibited by ONX 0914. In LMP7^−/−^ mice, the myeloid immune cell counts resemble those of WT mice, whereas ONX 0914 exerts distinct effects.

### Immunoproteasome inhibition by ONX 0914 during the second wave of CV infection fails to improve the cardiac output

Others and we have demonstrated the critical role of inflammatory monocytes as a source of pro-inflammatory and pro-fibrotic cytokines in the pathogenesis of myocarditis and its sequela, inflammatory cardiomyopathy [33, 35]. However, based on the different influences of ip depletion in LMP7^−/−^ mice and ip inhibition by ONX 0914 on CV myocarditis, it is unclear whether inhibitors of the ip, such as ONX 0914, would be effective in a clinically relevant setting to dampen adverse inflammatory responses during acute viral myocarditis. The established pro-inflammatory role of the ip leading to cardiac dysfunction in CV-infected A/J mice [2] prompted us to ask how ONX 0914 treatment, initiated after the first inflammatory wave seen 2–3 days after viral infection, can influence cardiac performance and the severity of viral myocarditis.

**Fig. 5:**
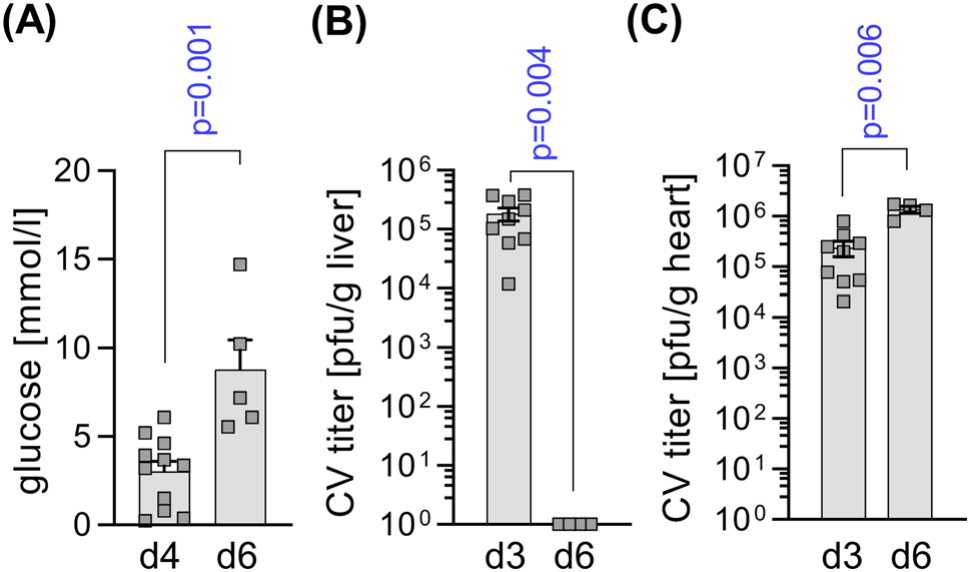
Biphasic course of CV infection in A/J mice. A/J mice were infected with CV Nancy. Mice were analysed on day 3/4 and day 6 after infection for **a** blood glucose levels using a clinically approved glucose meter and virus concentration in **b** liver and **c** heart tissue by plaque assay

**Fig. 6:**
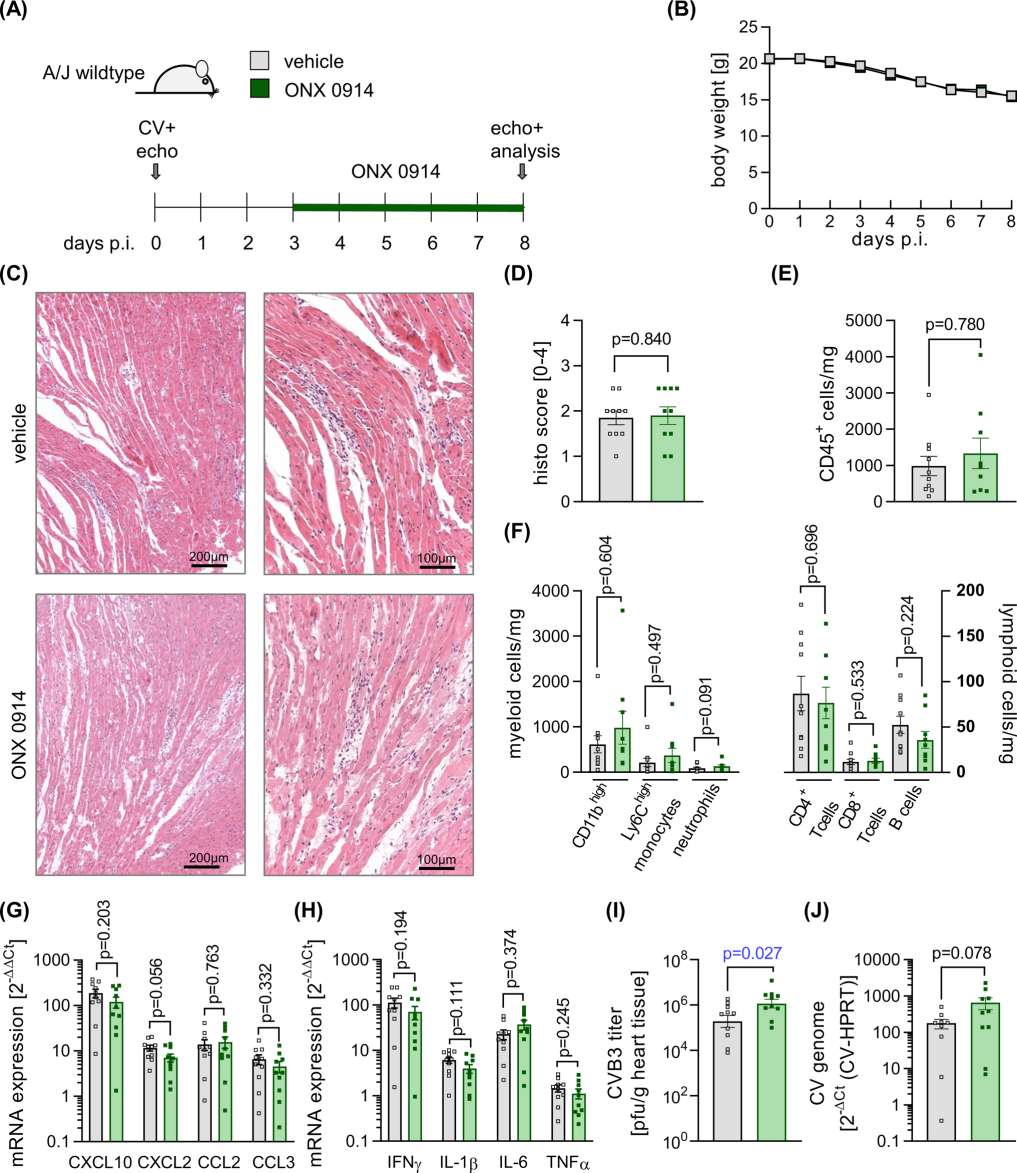
Impact of ONX 0914 treatment on viral myocarditis after systemic inflammation has been established. **a** Age and gender matched A/J mice were infected with CV Nancy. Starting on day 3, CV infected mice were either treated with the vehicle Captisol^®^ or with 10 mg/kg BW ONX 0914 daily (*n* = 10 per group) and mice were analysed on day 8. **b** Body weight was monitored daily. **c**, **d** Formalin fixated and paraffin embedded heart tissue sections were stained with hematoxylin and eosin and myocarditis severity was assessed. Representative micrographs of the hematoxylin and eosin stained heart tissue sections are depicted **d**. **e**, **f** Immune cell infiltration into the heart was quantified by flow cytometry. Chemotactic **g** and **h** pro-inflammatory cytokine mRNA expression in cardiac tissue on day 8 after infection, as determined by qPCR in both groups (*n* = 10) and normalized to vehicle (*n* = 5) or ONX 0914-treated control groups (*n* = 6). Virus concentration was quantified by plaque assay (I) and viral genome was analyzed by qPCR **j**. Data is summarized as mean ± SEM

To set up an appropriate experimental ONX 0914 treatment protocol for the second inflammatory wave with acute myocarditis in CV infected A/J mice, we considered the following aspects. We wished to initiate ONX 0914 treatment as soon as the primary inflammatory response due to affection of pancreas and liver tissue has been established, based on our previous experience with this model. As indicated recently for other laboratory mouse strains, CV infection of A/J WT mice triggers a hypoglycemic state with a prominent dip in serum glucose levels early after infection (Fig. 5a), in parallel with the peak systemic cytokine response around day three/four in A/J mice [2]. From this stage onward, the virus titer in the liver tissue drops, reaching a complete lack of infectious viral particles by 6 days after infection (Fig. 5b). The virus titer in heart tissue, however, increases at this point in time (Fig. 5c), reflecting active viral replication in cardiac tissue. Serum glucose levels serve as a surrogate parameter of the systemic inflammatory condition during infection and they rise again after 6 days (Fig. 5a). Based on these temporal aspects of established systemic inflammatory responses, 3 days after infection prior to virus replication in cardiac tissue, we initiated ONX 0914 in CV-infected A/J mice on day 3 and followed mice up to 8 days (Fig. 6a), where inflammatory responses peak in the heart. The overall weight loss that we observed during CV infection was not influenced by ONX 0914 treatment (Fig. 6b), with no CV-induced mortality, regardless of the treatment group (data not shown). Histological staining of cardiac sections revealed middle-grade necrosis/inflammation in both the ONX 0914 and Captisol-treated groups, with no indication of an anti-inflammatory response under the influence of ONX 0914 (Fig. 6c, d). This result was corroborated by quantitative analysis of immune cells in heart tissue by flow cytometry. The total number of CD45^+^ immune cells was equivalent in both treatment groups (Fig. 6e), with CD11b^high^ myeloid cells being the most abundant immune cells in the inflamed heart. Infiltration of inflammatory monocytes and neutrophils, but also that of T and B cells was not influenced by ONX 0914 (Fig. 6f). Similarly, the production of chemotactic cytokines as well as the production of pro-inflammatory cytokines, such as IFN-γ, IL-1β, IL-6 and TNF-α, was not affected by ONX 0914 treatment (Fig. 6h). The virus concentration as reflected by the viral genome count and viral particle load in heart tissue after 8 days, revealed a higher CV content in the ONX 0914 group (Fig. 6i, j).

**Fig. 7:**
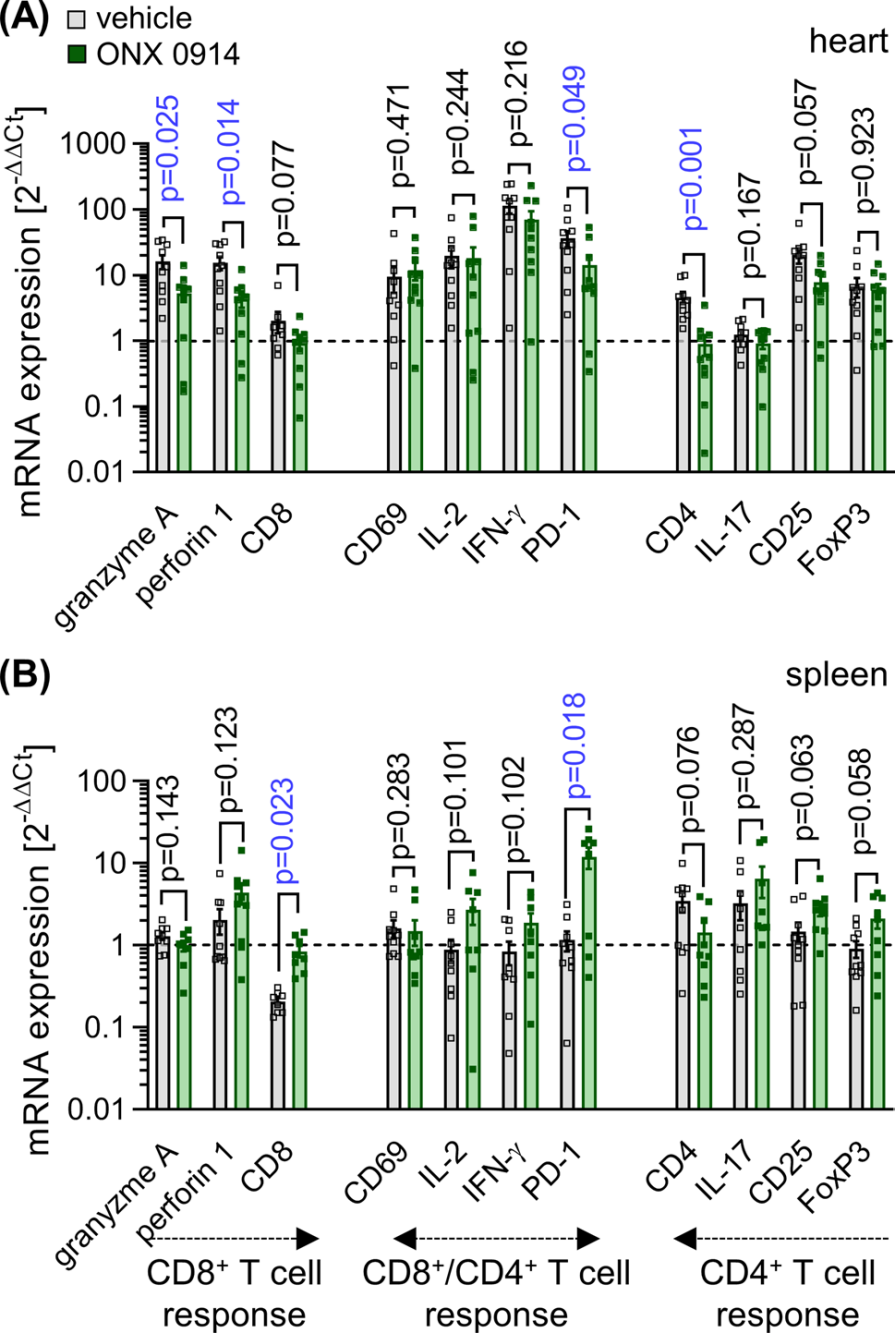
Impact of ONX 0914 treatment on T cell responses. Age and gender matched A/J mice were infected with CV Nancy. Starting on day 3, CV infected mice were either treated with the vehicle Captisol^®^ or with 10 mg/kg BW ONX 0914 daily (*n* = 10 per group) and mice were analysed on day 8. The data reflect cardiac **a** and splenic **b** mRNA expression levels, as determined by qPCR in each group (n = 10) and normalized to vehicle ( = 5) and *n*ONX 0914-treated control groups (*n* = 6). Data is summarized as mean ± SEM

Based on previous reports on re-shaped T cell differentiation with altered expression levels of inhibitory immune checkpoint molecules [12, 49], we then asked whether ONX 0914 treatment might influence the T cell response in viral myocarditis. We quantified the mRNA expression levels of the early activation antigen CD69, together with IL-2 and IFN-γ, for both CD8^+^ and CD4^+^ T cell activation, granzyme A and perforin 1 as effector molecules of a CD8^+^ T cell response, as well as IL17, CD25 and FoxP3 levels for CD4^+^ T cell differentiation, in addition to the immune checkpoint molecule PD-1. In infected heart tissue, IL-2 and IFN-γ levels, produced by both CD8^+^ and CD4^+^ T cells, were increased regardless of ONX 0914 treatment, a finding corresponding to equally elevated expression of CD69 and PD-1 (Fig. 7a). IL-17 production was not upregulated in infected heart tissue, which argues against a substantial infiltration of Th17 cells during viral myocarditis of A/J mice. The elevated expression levels of CD25 and FoxP3 in heart tissue indicated an infiltration of regulatory T cells, with no effect of ONX 0914 treatment. The quantification of granzyme A and perforin 1, both effector molecules of CD8^+^ T cells, revealed lower expression levels in ONX 0914-treated mice (Fig. 7a). This, in line with our previous study [2], indicates fewer active CD8^+^ T cells, a population making only a minor contribution to the pool of invading immune cells. Overall, our data show that ONX 0914 exerts no major effects on T cell responses in the infected heart. Since the heart tissue, as an effector organ of the adaptive immune response, might not reflect all systemic effects potentially exercised by ONX 0914, we also profiled CD8^+^ and CD4^+^ T cell activation in spleen, a secondary lymphoid organ (Fig. 7b). Splenic T cells had a lower activation status in comparison to the cells in heart tissue, as reflected by unaltered expression of CD69, IL-2, IFN-γ and granzyme A in infected mice, regardless of ONX 0914 treatment. Importantly, in the ONX 0914-treated group, mRNA expression profiling revealed a marked increase of FoxP3 and higher CD25 levels, indicative of an elevated presence of regulatory CD4^+^ T cells in comparison to the vehicle group. Together with elevated PD-1 levels, an inhibitory immune checkpoint molecule, these data indicate that ONX 0914 steers the adaptive CD4^+^ T cell response towards an immune suppressive activity, which leads to less effective control of the virus in heart tissue upon treatment with ONX-0914.

Nevertheless, despite the adverse effects of ONX 0914 on virus control, the inflammatory heart tissue damage during acute myocarditis was not substantially affected whenever blockade of ip activity was postponed to a time point after systemic inflammation was established. Importantly, the anti-inflammatory influence of ONX 0914 treatment observed when treatment was initiated prior to infection [2] vanished completely. Similar to our analysis of viral myocarditis in LMP7^−/−^ mice, echocardiography was performed to determine whether ONX 0914 treatment, applied during the second wave of infection, influences cardiac function. CV infection in A/J mice resulted in a significant drop of the cardiac output, mainly attributed to impaired left ventricular filling (Vol d), which was not influenced by ONX 0914 treatment (Table 2). The elevation of the LVEF in vehicle-treated mice during viral myocarditis, which was absent in the infected ONX 0914 group and had not been observed in any of our previous studies [2, 40], had virtually no effect on the cardiac output. We conclude that ONX 0914-mediated ip inhibition, initiated after the primary inflammatory response in CV infection has been established, cannot counteract the inflammation-triggered impairment of the cardiac output.

**Table 2:** Analysis of cardiac function in A/J mice after ONX 0914 treatment during acute CV myocarditis

	Vehicle		ONX 0914	
	Baseline	Day 8	Baseline	Day 8
Heart rate (bpm)	401 ± 11	354 ± 15	410 ± 10	379 ± 14
Trace EF (%)	48.6 ± 1.6	57.2 ± 3.3	47.6 ± 1.0	46.1 ± 3.1^§^
Cardiac output (mL/min)	8.9 ± 0.4	6.2 ± 0.3*	8.2 ± 0.3	5.9 ± 0.5*
Stroke volume (µL)	22.2 ± 1.0	17.4 ± 0.7*	20.0 ± 0.6	15.5 ± 1.3*
Vol d (µL)	45.6 ± 1.2	31.4 ± 2.1*	42.1 ± 0.8	33.5 ± 1.6*
Vol s (µL)	23.4 ± 0.8	14.0 ± 1.8*	22.1 ± 0.6	18.1 ± 1.3*^,§^
LVID-d (mm)	3.7 ± 0.1	3.3 ± 0.1*	3.7 ± 0.1	3.2 ± 0.1*
LVID-s (mm)	2.8 ± 0.1	2.4 ± 0.1*	2.8 ± 0.1	2.4 ± 0.1*

ONX 0914 (10 mg/kg BW) was initiated in A/J mice 3 days after to infection (*n* = 10). Echocardiography was performed prior to infection (baseline, day 0) and on day 8 post-infection. Age- and gender-matched vehicle-treated mice served as controls (*n* = 10 vehicle). Data shown are mean values ± SEM and were analyzed using repeated measurements two-way ANOVA, followed by Sidak’s multiple comparison test

EF: ejection fraction; bpm: beats per minute; Vol d/s: end-diastolic/-systolic left ventricular volume; LVID-d/s: left ventricular inner dimension at diastole/systole

*Indicates significant differences in the respective treatment group at day 8 compared to this groups’ baseline measurement. § indicates significant differences regarding to ONX 0914 treatment after infection

## Discussion

In a recent study, we demonstrated that, in contrast to the effects seen in CV-infected C57BL/6 and NMRI mice [2, 40], the ip inhibitor ONX 0914 protects A/J mice from developing CV myocarditis. In A/J mice, where the severe CV-triggered pathology is primarily attributed to a sepsis-like cytokine storm and distributive shock condition, ip activity is on the whole detrimental [2]. Unlike C57BL/6 mice [28], A/J mice can also be used as a model organism to investigate troponin I-directed cardiac autoimmunity [20]. AM is known to be triggered by viral infection [46] or by therapy with immune checkpoint inhibitors (ICI) targeting, e.g., the PD-1/PD-1L pathway [25], with ICI-related myocarditis being a severe, if rare, side effect in cancer immunotherapy [12]. Similar to viral myocarditis, in AM of A/J mice, the ip stimulates the production of chemotactic and pro-inflammatory cytokines, the later governing CD4^+^ T cell differentiation into Th17 and Th1 cells in autoimmune heart disease [39] and cumulating in cardiac tissue damage and dysfunction [12]. Based on these findings, we set up this study, aiming to obtain a deeper insight into the immune pathways that contribute to the adverse effects achieved by ip-mediated proteolysis in CV-infected A/J mice and simultaneously question the therapeutic utility of ip inhibitors for severe viral myocarditis. Altogether, our findings demonstrate that the therapeutic efficacy of ip inhibitors for CV-triggered myocarditis in A/J mice relies on their immunomodulatory effects on the systemic inflammatory response reaction.

Several aspects initially prompted us to investigate CV myocarditis in A/J-LMP7^−/−^ mice. From the mouse model of TnI-induced AM, we knew that the phenotype found in ip-deficient mice, seen for LMP2^−/−^ and LMP2^−/−^/LMP7^−/−^/Mecl-1^−/−^ mice in addition to LMP7^−/−^ mice, has the same mitigated inflammation during AM, and this was also observed in mice treated with the ip inhibitor ONX 0914 [12]. Contrary to the effects attributed to ip inhibition in this model, PRR-triggered pro-inflammatory cytokine responses, however, are not substantially affected by genetic ablation of ip subunits [11, 15, 37]. These divergent results, obtained with TLR-activated macrophages or monocytes from LMP7^−/−^ mice in comparison to ONX 0914-treated cells from WT mice, indicate that the elevated expression of the standard proteasome, seen, e.g., in spleen tissue of LMP7^−/−^ mice [12], triggers similar pro-inflammatory cytokine responses, as the ones mediated by ip proteolysis in WT mice. Other than that, a short-term ONX 0914 treatment, as shown here, has no relevant influence on the level of the standard proteasome after 24 h in cells from spleen or bone marrow. The reduction of the cardiac output in CV-infected LMP7^−/−^ mice, which is mainly attributed to lower ventricular filling and does not involve a deterioration of the systolic function of the left ventricle, is not altered in comparison to WT controls. Diastolic filling in CV-infected mice reflects the systemic pro-inflammatory reaction, leading to lower peripheral resistance of the vasculature [2, 13, 32]. The reduced cardiac output that we found in both CV-infected LMP7^−/−^ mice and littermate WT controls implies similar cytokine responses triggered by CV infection in both hosts. Moreover, this finding also implicates that the improved cardiac filling, which we reported earlier for CV-infected A/J mice under the influence of ONX 0914 [2], is at least partially attributed to a suppression of the systemic cytokine response early upon infection, found in infected animals with blocked ip activity and lack of compensatory standard proteasome formation [40].

Supportive data for the differential myeloid-cell derived inflammatory immune response in WT mice with ONX 0914-blocked ip activity in comparison to genetic ablation of ip subunits in LMP7^−/−^ mice comes from our finding that myeloid immune cell counts are heterogeneously affected. The prominent ONX 0914-mediated neutrophilia [2, 38], which is due to promoted egress of this cell population from the bone marrow niche [2], is less well developed, if it is present at all, in LMP7^−/−^ mice. Other than by ONX 0914 treatment, where consistent with findings of this study, reduced human monocyte counts emerge [6], monocyte counts in infected, but also in naive LMP7^−/−^ mice, show no differences relative to those measured in WT mice. From these experiments, we conclude that the pathophysiological effects controlled by inflammatory monocytes, which are crucial in CV myocarditis [35], might differ a great deal between ip-deficient LMP7^−/−^ mice and WT mice, where ip proteolysis is inhibited by ONX 0914 only for a short time. In LMP7^−/−^ mice, the myeloid immune cell counts resemble those of WT mice, whereas ONX 0914 exerts specific effects, indicating that the ip controls cellular processes, such as survival and/or proliferation of inflammatory monocytes [6]. In ip-deficient mice with inborne defects of LMP7, these effects are not observed, suggesting other genes are compensating for the missing gene products. Such biological compensation, attributed to increased standard proteasome expression in LMP7^−/−^ mice, apparently camouflages the contribution of ip activity to the pathology observed early in CV infection. Nevertheless, cells after long-term treatment with ONX 0914, such as for a 4 week period in AM, exert changes of the proteasome subunit expression profile similar to those seen in LMP7^−/−^ mice [12]. Such adaption by the standard proteasome counterpart of the ip explains why ONX 0914-mediated effects, such as on myeloid cell abundance, can vanish after such a prolonged treatment protocol.

Other than has been shown for the systemic inflammatory response, the impact of ip proteolysis on CD4^+^ T cell activation and differentiation towards Th17 lymphocytes with less regulatory T cells, as well as suppression of inhibitory immune checkpoint molecules, such as PD-1, appears to be the same for LMP7^−/−^ mice and ip-inhibitor treated animals [7, 9, 12, 37]. In fact, this study confirmed the role of ip inhibitors for steering CD4^+^ T cell differentiation towards a suppressive phenotype with more regulatory T cells and higher expression of PD-1 in spleen tissue, also during viral myocarditis. The finding that myocardial inflammation remains unaffected in both LMP7^−/−^ and ONX 0914-treated mice (days 3–8) indicates that those adaptive immune responses operative in troponin I AM, most likely do not substantially contribute to the manifestation of acute CV myocarditis in A/J mice. On the other hand, the immunosuppressive activity of ONX 0914 on pro-inflammatory CD4^+^ T cell immunity might partially explain why the virus concentration was increased in heart tissue under the influence of ONX 0914 (days 3–8). In troponin I AM, heart-directed autoimmunity in A/J mice results in reduced systolic function of the left ventricle, with mitigated inflammation in LMP7^−/−^ mice leading to improved cardiac function [12]. However, in viral myocarditis, others and we found no decrease in the contractile performance of the heart, as would be reflected by reduced LVEF, irrespective of the genetic background of the laboratory mouse strains tested [2, 34, 35, 40]. Therefore, even if a beneficial effect, such as ablation of LMP7, were to be expected, a biologically relevant improvement of the LVEF in CV-infected mice would be unlikely. Altogether, our experiments with CV infected LMP7^−/−^ (A/J) mice indicate that the pro-inflammatory action achieved by ip proteolysis is compensated for by an altered proteasome isoform pattern [12, 48] that virtually balances the biological effects seen in infected WT mice.

The anti-inflammatory capacity of ip inhibitors is remarkable in troponin I AM [12] and, similar to observations in experimental autoimmune encephalomyelitis (EAM) [9], also the progression of the disease can be ameliorated by ONX 0914, even after autoimmunity has been established in these models. In contrast to these reports on autoimmunity, where ONX 0914 is protective following both prophylactic and therapeutic approaches, in this study, we demonstrate that delay of the initiation of ONX 0914 treatment to a time point when systemic inflammatory responses in CV infected A/J mice emerge prior to cardiac injury, has no beneficial effect on viral myocarditis. In fact, reminiscent of previous reports in NMRI [40] and C57BL/6 mice [2], we found that ONX 0914 treatment apparently interfered with host response pathways, which limit the virus concentration in heart tissue. In addition to altered CD4^+^ T cell immunity, this might be attributed to a lack of ip selectivity for ONX 0914 in inflamed heart tissue, with the compound leading to substantial inhibition of the standard cardiac proteasome subunit β5 at this stage. Regarding the anti-inflammatory role of ONX 0914, we confirmed earlier that ONX 0914 efficiently blocked ip function also at advanced stages of CV infection, such as in spleen tissue [40], arguing against any loss of inhibitory capacity by ONX 0914 in immune cells with constitutive ip expression. Thus, the anti-inflammatory action of ip inhibitors must take place prior to the manifestation of the first inflammatory wave in A/J mice to exert a biologically relevant effect on CV infection. During CV myocarditis, the cardiac output, together with the diastolic filling, were not influenced by ONX 0914 applied between 3 and 7 days after infection. In contrast, ONX 0914-mediated inhibition of systemic inflammation in CV infection, whenever the compound was administered prior to infection, resulted in higher ventricular filling and improved cardiac output [2]. Based on this, we hypothesize that, in general, mainly the systemic inflammatory condition and the thereby altered vascular tonus contribute to the reduced cardiac output, seen after CV infection, whereas cardiac injury, if it has any influence at all, it is a minor one. Further support for the fact that the systemic inflammatory response is the main determinant for the reduced cardiac output in infected mice comes from another study. Infection of mice with a genetically engineered CV, exerting a comparatively mild first inflammatory wave, yet targeting the heart, develop no functional deterioration of the heart despite the emergence of viral cytotoxicity and inflammatory responses in the heart [44].

In conclusion, this study demonstrates that the beneficial effects to mitigate CV-triggered myocarditis in A/J mice due to inhibition of the ip rely on their immunomodulatory effects on the systemic inflammatory response reaction. To mitigate cardiac inflammation in viral myocarditis, blockade of the ip needs to be accomplished by the inhibitory compound, prior to when the cytokine-triggered global inflammatory response promotes cardiac inflammatory injury. Data from other laboratory strains, such as C57BL/6 and NMRI mice, obtained while undergoing CV myocarditis, showed that treatment with ONX 0914 either had no effect or it resulted in exacerbated myocardial inflammation [2, 40]. Together with these results, the need for targeting the systemic inflammatory response prior to the onset of myocardial injury to achieve mitigated viral myocarditis in A/J mice hampers the utility of ip inhibitors for acute viral heart disease.
